# Costs of medical evacuation and transportation of First Nations Peoples and Inuit who travel for medical care in Canada: A systematic review

**DOI:** 10.17269/s41997-024-00945-y

**Published:** 2024-10-11

**Authors:** Majd Radhaa, Jennifer Leason, Aisha Twalibu, Erin Davis, Claire Dion Fletcher, Karen Lawford, Elizabeth Darling, Lloy Wylie, Carol Couchie, Diane Simon, Ava John-Baptiste

**Affiliations:** 1 Department of Epidemiology and Biostatistics, Western University, London, ON Canada; 2 Department of Anthropology and Archaeology, University of Calgary, Calgary, AB Canada; 3 National Council of Indigenous Midwives, Montreal, QC Canada; 4 Midwifery Education Program, Toronto Metropolitan University, Toronto, ON Canada; 5 Department of Obstetrics and Gynecology, McMaster University, Hamilton, ON Canada; 6 Departments of Pathology, Psychiatry and Anthropology, Western University, London, ON Canada; 7 Interfaculty Program in Public Health, Western University, London, ON Canada; 8 Association of Ontario Midwives, Toronto, ON Canada; 9 Department of Anesthesia & Perioperative Medicine, Western University, London, ON Canada

**Keywords:** Indigenous, First Nations, Inuit, Midwifery, Obstetric evacuation, Cost, Autochtones, Premières Nations, Inuits, profession de sage-femme, évacuation obstétricale, coûts

## Abstract

**Objective:**

For First Nations people and Inuit who live on reserves or in rural and remote areas, a guideline requires their travel to urban centres once their pregnancy reaches 36–38 weeks gestation age to await labour and birth. While not encoded in Canadian legislation, this guideline—and invisible policy—is reinforced by the lack of alternatives. Research has repeatedly demonstrated the harm of obstetric evacuation, causing emotional, physical, and financial stress for pregnant and postpartum Indigenous women and people. Our objective was to describe the costs of obstetric evacuation, as reported in the literature.

**Methods:**

We conducted a systematic review using online searches of electronic databases (Ovid EMBASE, CINAHL, Ovid Healthstar, PubMed, ScienceDirect, PROSPERO, and Cochrane Database of Systematic Reviews) and identified studies that reported costs related to medical evacuation or transportation in rural and remote Indigenous communities. We performed critical appraisal of relevant studies.

**Synthesis:**

We identified 19 studies that met the inclusion criteria. The studies reported various types of cost, including direct, indirect, and intangible costs. Medical evacuation costs ranged from CAD $7714 to CAD $31,794. Indirect and intangible costs were identified, including lost income and lack of respect for cultural practices.

**Conclusion:**

Costs associated with obstetric evacuation are high, with medical evacuation as the most expensive direct cost identified. Although we were able to identify a range of costs, information on financing and funding flows was unclear. Across Canada, additional research is required to understand the direct costs of obstetric evacuation to Indigenous Peoples and communities.

## Résumé

**Objectif:**

Une ligne directrice oblige les personnes inuites et des Premières Nations vivant dans des réserves ou des régions rurales et isolées et qui en sont entre leur 36^e^ et leur 38^e^ semaine de grossesse à se rendre dans un centre urbain pour y attendre le travail et l’accouchement. Bien qu’elle ne soit pas enchâssée dans la loi canadienne, cette ligne directrice (et le principe qu’elle cache) est renforcée par l’absence de solutions de rechange. Des études ont démontré à maintes reprises les préjudices de l’évacuation obstétricale, qui cause un stress émotionnel, physique et financier pour les femmes et les personnes enceintes autochtones en période postpartum. Nous avons cherché à décrire les coûts de l’évacuation obstétricale figurant dans la littérature spécialisée.

**Méthode:**

Nous avons mené une revue systématique en consultant des bases de données électroniques (Ovid EMBASE, CINAHL, Ovid Healthstar, PubMed, ScienceDirect, PROSPERO et Cochrane Database of Systematic Reviews), puis en répertoriant les études faisant état des coûts de l’évacuation médicale ou du transport médical dans les communautés autochtones rurales et éloignées. Nous avons ensuite effectué une évaluation critique des études pertinentes.

**Synthèse:**

Dix-neuf études répondaient aux critères d’inclusion. Elles faisaient état de divers types de coûts : directs, indirects et intangibles. Les coûts de l’évacuation médicale variaient de 7 714 $ à 31 794 $ CAN. Les coûts indirects et intangibles identifiés étaient la perte de revenu et le manque de respect pour les pratiques culturelles.

**Conclusion:**

Les coûts associés à l’évacuation obstétricale sont importants, et le coût direct le plus élevé est celui de l’évacuation médicale. Nous avons été en mesure de cerner une fourchette de coûts, mais les informations sur le financement et les flux de financement n’étaient pas claires. Partout au Canada, il faudrait pousser la recherche pour connaître les coûts directs de l’évacuation obstétricale pour les personnes et les communautés autochtones.

## Introduction

### Indigenous Peoples across Canada

Indigenous Peoples is an umbrella term used across Canada to describe the original peoples of North America, the land referred to by some Indigenous Peoples as Turtle Island. The terminology used to describe Indigenous Peoples in the territory known today as Canada has continually evolved. “Indigenous Peoples” in Canada typically refers to three groups of Indigenous Peoples recognized by the Canadian Constitution: First Nations, Inuit, and Métis (Crown-Indigenous Relations & Northern Affairs Canada, 2022). For Indigenous Peoples across Canada, there is a long history of community-based birthing and midwifery (National Council of Indigenous Midwives (NCIM), 2019, 2023). Colonial approaches—such as ongoing medicalization and systemic racism in the Canadian health care systems—undermine Indigenous strengths-based practices. These colonial approaches have a negative psychosocial impact, and we anticipate that they are also economically costly. Part of the reason these costs are underestimated is the jurisdictional overlap, which involves both cost sharing and cost shirking (Wylie et al., 2019).

### Indigenous Peoples and health care across Canada

Varying levels of government share a degree of jurisdiction and responsibility for delivering health care to Indigenous Peoples across Canada, which include First Nations, Inuit, and Métis. The Canada Health Act “requires that all medically necessary hospital, physician and surgical dental services be covered by provincial or territorial health care insurance plans for all eligible residents of the province or territory, including Indigenous Peoples” (Indigenous Services Canada, 2021). In addition to the Canada Health Act, a complex web of legislation, treaties, policy, and integrated agreements expands on Indigenous Peoples’ rights to health care across Canada (Craft & Lebihan, 2021; Indigenous Services Canada, 2021; Lavoie et al., 2011).

The federal government is responsible for providing direct delivery of health care services to First Nations people and Inuit across Canada, which is carried out through the First Nations and Inuit Health Branch (FNIHB) (Indigenous Services Canada, 2023). The health care plan known as the Non-Insured Health Benefits (NIHB) program is administered and managed by FNIHB. NIHB covers medical and related expenses, and can be utilized by any First Nations individual who is registered according to the Indian Act (also known as “Status Indian”) and any Inuk recognized by an Inuit land claims organization (Indigenous Services Canada, 2021, 2022). Currently, NIHB are not offered to Métis; as such we have not included Métis in our review. NIHB coverage expands beyond its provincial or territorial counterparts. NIHB coverage includes transportation, medical evacuation/transportation, meals, and accommodations which represent the cost categories of interest for this review. Notably, other NIHB coverage also includes a specific formulary of pharmaceuticals, medical supplies and equipment, dental benefits, vision care, and mental health counselling. As of March 3, 2021, there were 898,839 people eligible for coverage under the NIHB program (Indigenous Services Canada, 2022, 2023).

### Non-Insured Health Benefits expenditures

Between April 1, 2020, and March 31, 2021, the NIHB program benefits expenditure was CAD $1491 million. Medical transportation was the second highest expenditure category, behind pharmacy expenditures at 35.3% and 37% of total expenditures, respectively (Indigenous Services Canada, 2022). Historically, these are the top two expenditure categories.

### Obstetric evacuation

Obstetric evacuation is a policy that requires First Nations and Inuit people who live on reserves or in rural and remote areas to travel to urban hospitals for birth. While not encoded in Canadian legislation, the policy is specified in clinical practice guidelines and is reinforced by the lack of alternatives (FNIHB, 2011; Jumah et al., 2021; Kaufert & O’Neil, 1990; Lawford, 2016; Lawford & Giles, 2012; Lawford et al., 2018, 2019). For First Nations people and Inuit, the FNIHB Clinical Practice Guidelines for Nurses in Primary Care outlines the requirement to “arrange for transfer to hospital for delivery at 36‒38 weeks’ gestational age according to regional policy.” The latest publicly available version of the obstetrics chapter guideline was last revised in July 2011 (FNIHB, 2011). In practice, high-risk pregnancies can lead to evacuation and increased travelling distance to reach adequately equipped delivery units in critical care hospitals. In most communities, obstetric evacuation is the only option that is deemed to satisfy safety concerns. This highlights the pressure on both provider and patient to accept the status quo, and evacuate even in cases of low-risk birth. Exploring alternative models of care, such as Indigenous midwifery, can support both patients and care providers. Research has demonstrated the harm of this obstetric evacuation policy, causing emotional, physical, and financial stress for pregnant and postpartum Indigenous people (Brown et al., 2011; Chamberlain & Barclay, 2000; Kolahdooz et al., 2016; Lawford et al., 2019; Lee et al., 2022; Moffitt & Robinson Vollman, 2006; O’Neil et al., 1988; Sennett & Dougherty, 1991; Silver et al., 2022). The obstetric evacuation “guideline” is commonly referred to, and regarded as, the obstetric evacuation policy in the literature.

The motivation for this systematic review stemmed from the lack of descriptive analysis of the costs of obstetric evacuation in the literature. The disjointed administration and delivery of health care is partially attributable to the lack of comprehensive assessment of costs of obstetric evacuation. This systematic review examined various types of cost, including direct, indirect, and intangible costs. The main costs identified pertained to evacuation and transportation associated with medical care.

### Cost analysis framework

Several frameworks exist for describing and categorizing the cost of health services. We opted to categorize costs using a framework based on the cost of illness (CoI) methodology, proposed by Rice et al. (1985). This commonly used framework divides costs into direct, indirect, and intangible costs. Direct costs are “the value of resources that could be allocated to other uses in the absence of disease” while indirect costs are “the value of lost output because of cessation or reduction of productivity caused by morbidity and mortality” (Rice et al., 1985). Within this framework, Rice et al. did not attempt to measure intangible costs such as pain and suffering. The intangible costs are assumed to be “unquantifiable.” We chose this framework to report costs in a way recognizable to health economists. However, we acknowledge that the cost analysis framework may not reflect Indigenous ways of knowing and being. For example, Indigenous people may not consider “intangible” costs an appropriate way to describe feelings of fear and anxiety or the loss of birth knowledge associated with obstetric evacuation.

### Economic cost of medical evacuation and transportation

To date, there has been no attempt to estimate the full scope of the costs associated with obstetric evacuation. Although FNIHB provides annual reports that include total direct costs associated with medical evacuation and transportation, these reports do not provide a comprehensive, detailed enumeration of the cost items.

## Methods

### Search strategy and search design

We developed a search strategy in consultation with an information specialist with expertise in Indigenous health (see Appendix). We searched the following databases: Ovid EMBASE (1947–2022), CINAHL (1981–2022), Ovid Healthstar (1966–2022), PubMed, ScienceDirect, PROSPERO, and Cochrane Database of Systematic Reviews. This systematic review has been registered with the International Prospective Register of Systematic Reviews (PROSPERO) database under the registration number CRD42022353800.

### Inclusion and exclusion criteria

Study inclusion criteria were economic evaluation studies of all types, including cost-effectiveness, cost–benefit and cost-utility analyses, descriptive cost analyses, economic cost models, economic evaluations, randomized control trials including quasi-randomized and cluster-randomized trials, observational studies, case studies, and relevant qualitative studies that reported costs.

Study exclusion criteria were: studies without assessment of costs, studies measuring/estimating fertility rates and/or evacuation rates without cost parameters, studies outside Canada, studies in urban settings, studies focused on Métis or non-Indigenous people, and studies focused on medical evacuation from military bases.

Medical evacuation and transportation, inclusive of all transportation modes, were considered. The search was inclusive of transportation by air (medevac, plane, helicopter), transportation by land (car, train, bus, taxi, snowmobile), and transportation by waterways (ferry, boat).

Two authors (MR, AT) independently screened citations and full texts of manuscripts. Disagreements were resolved through consensus.

### Data extraction

Data were extracted using a data extraction form in Covidence, which included lead author, year of publication, community/region in which study was conducted, study objective, start and end dates of study, funding sources, conflicts of interest, population description, number of participants, study inclusion and exclusion criteria, recruitment method, cost items, cost item currency, cost item category, cost item description, and relevant quotes. The community/region was defined using the name and/or location conventions used in each respective article. This included specific First Nations (e.g. Black Lake First Nation), geographic location (e.g. Puvirnituq, along the eastern coast of Hudson Bay), or a combination of both (e.g. Indigenous northern communities across Canada). Cost items were assigned to one of the following cost categories: a direct cost (e.g. cost of a flight in Canadian dollars), indirect cost (e.g. missed work and associated lost income), or an intangible cost (e.g. stress). We abstracted all relevant cost items, which included medical evacuation, incentive grants for physicians, transportation, accommodation, meals/food, out-of-pocket expenses, and ambulance services. Costs were adjusted for inflation to 2022 Canadian dollars using the Canadian Consumer Price Index (CPI) (Statistics Canada, 2023).

Intangible costs represent real and impactful consequences of the obstetric evacuation policy even though these costs were not assigned a monetary value. We abstracted direct quotes from the included studies, to honour the voices and experiences of the study participants who have been impacted by obstetric evacuation. Some quotes from authors of included studies were also abstracted, when they summarized reoccurring sentiments shared by study participants. We grouped the intangible costs into themes, basing the categories on the quotes.

Data abstraction was performed by one person (MR) and verified by another (AT). We used the Covidence software to manage the systematic review data (Covidence systematic review software, 2022).

## Synthesis

### Number of studies identified

**Fig. 1:**
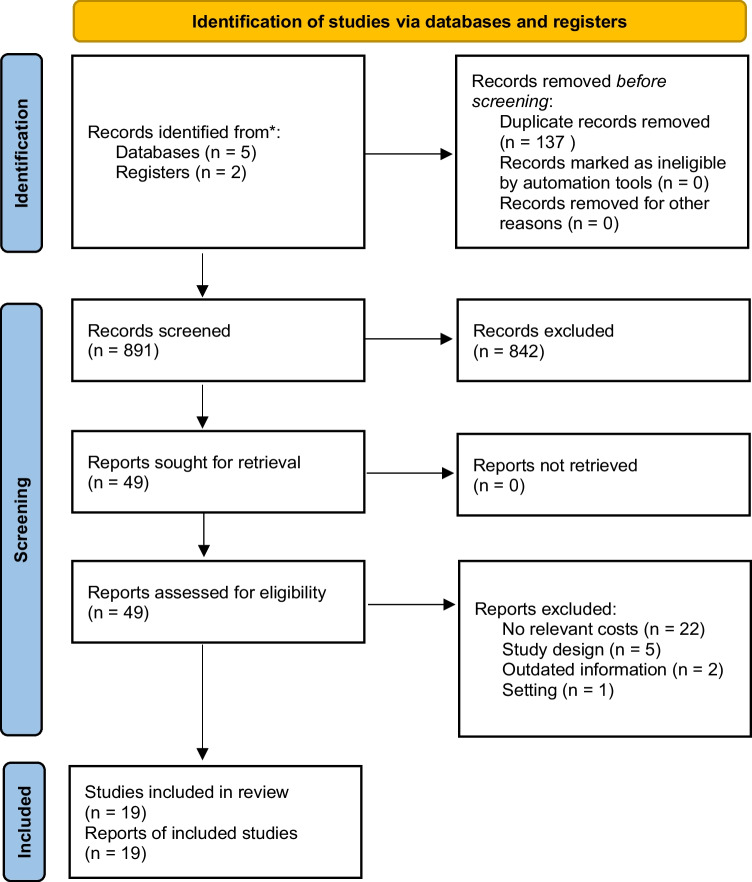
PRISMA 2020 flow diagram for new systematic reviews which included searches of databases and registers only

Figure 1 illustrates the Preferred Reporting Items for Systematic reviews and Meta-Analyses (PRISMA) flow diagram of the identified studies (Page et al., 2021). The electronic database search resulted in accumulation of 49 full-text articles assessed for eligibility. A total of 30 studies were excluded and a total of 19 articles were identified as appropriate for synthesis.

### Screening

In total, 1028 records were retrieved from the databases. After removing duplicates, 891 records underwent level 1 screening, in which we reviewed titles and abstracts. Of these, 49 records were assessed for eligibility using level 2 screening, in which we retrieved and reviewed the full text of each study. From an initial total of 49 eligible studies, 22 studies were excluded as they did not include relevant costs, five studies were excluded due to the study design, two were excluded for presenting outdated information or due to retractions, and one record was excluded due to an irrelevant setting. Therefore, a total of 19 studies were included in the qualitative synthesis.

### Study characteristics

The characteristics of included studies are summarized in Table 1. Research outlining the costs of obstetric evacuation was published as early as 1988, with modern research today highlighting the persistent gaps in understanding the economics of obstetric evacuation. The studies pertained to various First Nations and Inuit communities across different regions across Canada. Three studies took place in British Columbia; three in Manitoba; four in Keewatin Region, Northwest Territories (today known as Kivalliq, Nunavut); two in the Northwest Territories; two in Quebec; one in Saskatchewan; one in Ontario; and one in Nunavut. Two studies did not specify an exact location or community.

**Table 1:** Included studies characteristics

Author, year	Title	Community/region in which the study was conducted	Aim/goal of study	Study design
(Adams et al., 2021)	Access to ultrasound imaging: A qualitative study in two northern, remote, Indigenous communities in Canada	Stony Rapids and Black Lake First Nation Saskatchewan, Canada	To explore perceptions of access, and factors which shape access, to ultrasound imaging in 2 northern, remote, Indigenous communities in Canada	Qualitative research
(Brown et al., 2011)	The birthing experiences of rural Aboriginal women in context: implications for nursing	Haida, Kwakwa-ka- ‘wakw, and Nuxalk First Nations British Columbia, Canada	To contribute to rural maternity care in Aboriginal communities by exploring birthing experiences and traditional birthing practices amongst the Haida, Kwakwa-ka- ‘wakw, and Nuxalk First Nations	Qualitative research
(Cardinal, 2018)	“Lost births,” service delivery, and human resources to health	Indigenous northern communities in Canada	To advocate for improved service delivery of maternal-newborn care in northern Indigenous communities	Literature review
(Chamberlain et al., 1998)	Evaluation of a midwifery birthing center in the Canadian north	Rankin Inlet, Hudson Baker Lake, Keewatin Region Nunavut, Canada	To identify whether it would be safe, cost-effective, and psychologically and socially satisfying for Inuit women in 1 community in the Northwest Territories	Evaluative study using quantitative and qualitative methods
(Chamberlain & Barclay, 2000)	Psychosocial costs of transferring indigenous women from their community for birth	Keewatin Region, Central Canadian Arctic	To describe the psychosocial effects of transferring Canadian Inuit women out of their communities for birth	Qualitative research
(Chamberlain et al., 2001)	Aboriginal birth: psychosocial or physiological safety	Canadian Arctic Northwest Territories, Canada	To raise awareness and stimulate discussion and research into maternity care options for Aboriginal women living in remote areas	Evaluative study using quantitative and qualitative methods
(Chatwood, 1996; Chatwood-Affleck et al., 1998)	Indications for transfer for childbirth in Inuit women at the Innuulisivik Maternity	Povungnituk, along the eastern coast of Hudson Bay Quebec, Canada	To describe the indications for transfer of expectant Inuit women served by the Innuulisivik Maternity to secondary and tertiary (levels II and III) obstetrical centres in Iqaluit or Montreal	Descriptive study
(Cooper et al., 2021)	Patient healthcare experiences in the Northwest Territories, Canada: an analysis of news media articles	Yellowknife Northwest Territories, Canada	To examine the characteristics of patient health care experiences as reported through news media in the Northwest Territories	Case series; literature review
(Kornelsen & Grzybowski, 2005)	Safety and Community: The Maternity Care Needs of Rural Parturient Women	7 rural communities in Canada	Investigate rural parturient women’s experiences of obstetric care in the context of the social and economic realities of life in rural, remote, and small urban communities	Qualitative research
(Kornelsen & Grzybowski, 2006)	The Reality of Resistance: The Experiences of Rural Parturient Women	British Columbia, Canada	To explore these issues with women from 4 rural British Columbian communities through semi-structured interviews and focus groups	Qualitative research
(Lawford et al., 2018)	Canada’s evacuation policy for pregnant First Nations women: Resignation, resilience, and resistance	Manitoba, Canada	To contribute scholarship that describes First Nations women’s and community members’ experiences and perspectives of Health Canada’s evacuation policy in Manitoba	Qualitative research
(Lawford et al., 2019)	“This policy sucks and it’s stupid:” Mapping maternity care for First Nations women on reserves in Manitoba, Canada	Manitoba, Canada	Present new and detailed information about Canada’s health policy as well as recommendations to address the health care gaps identified	Qualitative research
(Lee et al., 2022)	Returning childbirth to Inuit communities in the Canadian Arctic	Kivalliq Nunavut, Canada	Examine why birthplace choice has not been given back to Inuit yet	Literature review
(Milnes et al., 1993)	A retrospective analysis of the costs associated with the treatment of nursing caries in a remote Canadian aboriginal preschool population	Manitoba, Canada	Analyze costs associated with the treatment of nursing caries in Indigenous children who live in remote regions of Manitoba	Cohort study
(O'Driscoll et al., 2011)	Delivering away from home: the perinatal experiences of First Nations women in northwestern Ontario	Northwestern Ontario, Canada	To understand the perinatal knowledge and experiences of First Nations women from northwestern Ontario who travel away from their remote communities to give birth	Qualitative research
(O'Neil et al., 1988)	Inuit concerns about obstetric policy in the Keewatin region, N.W.T	Keewatin Region Northwest Territories, Canada	To evaluate policy options for perinatal health services in Inuit communities	Qualitative research
(O'Neil et al., 1991)	Obstetric policy for the Keewatin Region, N.W.T.: results of the childbirth experience survey	Keewatin Region Northwest Territories, Canada	To document the problems created for Inuit women by the current obstetric policy which requires that all women leave their home communities for childbirth in Manitoba hospitals	Cohort study
(Van Wagner et al., 2007)	Reclaiming birth, health, and community: midwifery in the Inuit villages of Nunavik, Canada	Nunavik Quebec, Canada	Describe the Inuulitsivik midwifery service and education programs	Case report
(Varcoe et al., 2013)	Help bring back the celebration of life: a community-based participatory study of rural Aboriginal women’s maternity experiences and outcomes	Nuxalk, Haida and 'Namgis First Nations British Columbia, Canada	Understand rural Aboriginal women’s experiences of maternity care and factors shaping those experiences	Cohort study

Early research of obstetric evacuation focused on Inuit communities, particularly in the Keewatin Region, which was a jurisdiction of the Northwest Territories. With the creation of Nunavut in 1999, the region became part of the newly established territory, and is more commonly referred to today as the Kivalliq Region. Of the 19 studies, 17 were specifically focused on obstetric evacuation, encompassing topics such as ultrasound imaging, birth and delivery, Indigenous midwifery, transfer and evacuation, and broader maternity care needs. Of the two remaining studies, one conducted an analysis of news media articles assessing overall patient health care experiences in the Northwest Territories and the other conducted a retrospective analysis of costs associated with treating nursing caries in Manitoba. Although the aforementioned study’s focus was dental care rather than obstetric care, it met the inclusion criteria, as it included a cost evaluation of transportation for First Nations people travelling to reach care in an urban centre. Two literature reviews presented cost data not extracted from literature, rather, the costs were from other sources such as expert opinion and news articles that complemented the literature search findings of those respective studies. Qualitative research was the most common study design, with interviews as the main method of data collection. Other study designs such as evaluative studies, case series, and cohort studies were also included.

### Direct costs

Studies reported direct costs (Table 2) and when information was available, we categorized costs according to CADTH guidelines into direct costs incurred by the publicly funded health care payer, broader government payer, and costs to patients and informal caregivers. Studies that reported the payer identified a variety of entities, including the federal government, provincial/territorial government, municipality, and band government. The most commonly reported costs were related to medical evacuation, transportation, and accommodation. Some studies reported transportation and accommodation as a combined cost, without itemizing the costs. Other reported costs included meals/food, out-of-pocket costs, ambulance services, and incentive grants for physicians. Some studies reported total costs while others reported per diems for a given cost item. Reported medical evacuation costs started at $7,714 for a one-way medical evacuation flight from regional communities to Puvirnituq, Quebec. Medical evacuation costs reached $31,794 for a medical evacuation flight from Rankin Inlet, Nunavut, to Yellowknife, Northwest Territories. Combined costs of transportation and accommodation costs varied greatly, with costs as low as $162 and reaching up to $11,524. Transportation costs reported independently of other costs ranged between $580 and $2,415, excluding co-pays and per diems. The large variation can be partially attributed to the geographic locations, as travel from remote communities seemed to present higher costs compared to travel from rural communities. None of the studies reported costs of accommodations independently, as they were either combined with transportation costs or reported as per diems. Per diem costs reported ranged from $25 to $125, covering mostly food/meals and accommodation. Accommodation per diems varied between those staying in private accommodation and with family or friends. Costs were adjusted for inflation to 2022, and reported in Canadian dollars.

**Table 2:** Direct costs of evacuation and transportation to reach and access medical care

Author, Year of publication	Number of participants	Currency year*	Cost (CAD)	Cost (CAD), CPI 2022	Cost item	Description
(Cardinal, 2018)	None; literature review	2016	$27,000	$31,794	Medical evacuation	Cost of a single medical evacuation flight from Rankin Inlet, NU, to Yellowknife, NWT (around 1140 km, 2 h by plane)
		2013	$80,000–116,600	$98,501.62–$143,566.12	Other: incentive grant for physicians	Value of incentive grant for physicians to set up practice in Northern Ontario. Distributed over 4 years
(Chamberlain & Barclay, 2000)	28	1998	$10,000	$16,561	Medical evacuation; transportation	Cost of medical evacuation/transportation to give birth away from home for 4 children
(Chatwood, 1996; Chatwood-Affleck et al., 1998)	411	1990	$4,000	$7,714	Medical evacuation	Cost of one-way medevac from surrounding communities to Povungnituk
		1990	$10,000	$19,286	Medical evacuation	Cost of one-way medevac from community via Kuujuarapik or Kuuiiuak to Montreal
(Lawford et al., 2018)	12	2016	$2,000	$2,355	Accommodation; meals/food; out-of-pocket expenses	Cost of one-way trip from reserve to Winnipeg, along with cost of accommodation, meals/food, and other expenses
(Lawford et al., 2019)	32	2017	$80–$96	$92.76–$111.31	Transportation	Cost of one-way trip for delivery for a mother travelling from Rankin Inlet to Winnipeg
		2017	$500	$580	Transportation; other: ambulance	Cost of one-way ambulance cost; according to a municipal staff member, the costs associated with an ambulance ride are payable to the City of Winnipeg. If the woman cannot afford to pay the price of an ambulance, which is approximately $500, the city absorbs the costs
		2017	$25–$50	$28.99–$57.98	Meals/food	Per diem of $25–$50 per day per patient to cover meals
(Lee et al., 2022)	None; literature review	2014	$14,000	$16,907	Transportation	Cost for a father and children to travel from Keewatin to Churchill
		2014	$15,000	$18,115	Medical evacuation	Cost of one-way medevac flight from Rankin Inlet to Winnipeg
		2014	$2,000	$2,415	Transportation	Cost of one-way return flight from Winnipeg to Rankin Inlet
		2022	$250	$250	Transportation	Co-pay fee for return airfare paid for by Nunavut Health Care Plan (NHCP); covered by those eligible for Non-Insured Health Benefits (NIHB)
		2022	$50–$125	$50–$125	Accommodation	Per diem of $50 per day for both patient and escort for stay in private accommodation
		2022	$50	$50	Meals/food	Per diem of $50 per day per person (patient and escort) for meals
(Milnes et al., 1993)	884	1984	$406	$1,013	Transportation; accommodation	Mean travel/lodging includes costs for air, bus, or automobile transportation and room and board costs for 1 adult and 1 child. Length of stay varied. Table 4, band group 1
		1984	$752	$1,876	Transportation; accommodation	Mean travel/lodging includes costs for air, bus, or automobile transportation and room and board costs for 1 adult and 1 child. Length of stay varied. Table 4, band group 2
		1984	$1,147	$2,862	Transportation; accommodation	Mean travel/lodging includes costs for air, bus, or automobile transportation and room and board costs for 1 adult and 1 child. Length of stay varied. Table 4, band group 3
		1984	$536	$1,337	Transportation; accommodation	Mean travel/lodging includes costs for air, bus, or automobile transportation and room and board costs for 1 adult and 1 child. Length of stay varied. Table 4, band group 1. Table 4, band group 4
		1984	$273	$681	Transportation; accommodation	Mean travel/lodging includes costs for air, bus, or automobile transportation and room and board costs for 1 adult and 1 child. Length of stay varied. Table 4, band group 1. Table 4, band group 5
		1984	$314	$783	Transportation; accommodation	Mean travel/lodging includes costs for air, bus, or automobile transportation and room and board costs for 1 adult and 1 child. Length of stay varied. Table 4, band group 1. Table 4, band group 7
		1984	$131	$327	Transportation; accommodation	Mean travel/lodging includes costs for air, bus, or automobile transportation and room and board costs for 1 adult and 1 child. Length of stay varied. Table 4, band group 1. Table 5, band group 6
		1984	$96	$240	Transportation; accommodation	Mean travel/lodging includes costs for air, bus, or automobile transportation and room and board costs for 1 adult and 1 child. Length of stay varied. Table 4, band group 1. Table 5, band group 8
		1984	$65	$162	Transportation; accommodation	Mean travel/lodging includes costs for air, bus, or automobile transportation and room and board costs for 1 adult and 1 child. Length of stay varied. Table 4, band group 1. Table 5, band group 9
		1984	$124	$309	Transportation; accommodation	Mean travel/lodging includes costs for air, bus, or automobile transportation and room and board costs for 1 adult and 1 child. Length of stay varied. Table 4, band group 1. Table 5, band group 10
		1984	$156	$389	Transportation; accommodation	Mean travel/lodging includes costs for air, bus, or automobile transportation and room and board costs for 1 adult and 1 child. Length of stay varied. Table 4, band group 1. Table 5, band group 11
(O'Neil et al., 1988)	N/A	1986	$5,000	$11,524	Transportation; accommodation; meals/food; out-of-pocket expenses	Cost of spouse and dependents (father and children) to accompany expecting mother in Churchill

^*^Currency year is based on mid-point of study time-frame. When study time-frame was not available, the currency year was calculated as the publication year minus 2

### Indirect costs

Studies also reported indirect cost (Table 3). These costs impacted the people evacuated, and in several instances impacted their partner, family, and community. The most common indirect cost was lost income due to travel. Other indirect costs included cost of childcare, cost of eldercare, lost land-based resources or income, and learning loss. The loss of income due to missed work was a recurring theme, especially in cases where the pregnant woman is the primary income provider for a family or household. Instances of partners missing work to care for children, leading to loss of income or of land-based resources, were also reported. If a partner travels with the pregnant person to provide support, that leads to further costs, including the direct costs of transportation and accommodation and indirect costs associated with missing work or arranging for childcare or eldercare. Since 2017, the federal government has provided coverage for the direct costs of “escort” travel (e.g. partner, mother), but the indirect costs are still incurred by the person evacuated and their family. (The Canadian Press, 2017). As indicated by Kornelsen and Grzybowski (2006) “One participant noted that her partner was off work for 12 days and went on to say, ‘That amount of time was hard because his was the only income coming in’.” The number of days obstetric evacuation keeps the expecting mother away from her community varies, and can span several weeks.

**Table 3:** Indirect cost and productivity loss due to travel to access medical care

Author, Year	Productivity cost	Quote
(Cardinal, 2018)	Lost income; cost of childcare	“Nonetheless, having a funded support person at the bedside does not necessarily address lost income or childcare issues, especially if that support person is the pregnant woman’s partner.”
(Chamberlain & Barclay, 2000)	Lost income; lost land-based resource(s)	“There was the additional cost of airfare if the partner came out too and there was the cost of the partner’s time off work or away from hunting to look after children: ‘Leaving the settlement is hard when you have children because you either have to try and afford to take your children out with you or have to rely on a sitter. And, if you are gone for a long time, it’s not fun especially if you are not with them or if you have to stay somewhere else, because you are not at home.’ (Mother A)” “One woman said that her husband was unfamiliar with housekeeping and therefore spent more than they could afford on food: ‘It is very expensive. The groceries at home are very expensive. It is hard for my husband to go out hunting because he has to take care of the kids. He usually goes out hunting to feed the kids but with no one to take care of the kids, it’s hard for him to go out hunting. And, with him not knowing how to cook very well, I think he was spending a lot of money on groceries for very expensive food, such as frozen TV dinners.’ (Mother C)”
(Chamberlain et al., 2001)	Lost income	“There was the additional airfare cost if the partner came out and the cost of his time off work. One woman said her husband was unfamiliar with housekeeping and therefore spent more than they could afford on food.”
(Chatwood, 1996; Chatwood-Affleck et al., 1998)	Lost income; lost land-based resource(s)	“If other family members do not help with childcare during a mother’s absence, men’s hunting and other work is disrupted with some husbands coming to resent women for going away.”
(Kornelsen & Grzybowski, 2005)	Lost income	“These [financial costs] included the travel expenses, long-distance telephone calls, and intrapartum transfer by ambulance, as well as partner’s lost income.”
(Kornelsen & Grzybowski, 2006)	Lost income	“Beyond explicit costs, many participants also acknowledged the cost of missed work their partners incurred to come with them to the referral community, even if only at the time of birth. One participant noted that her partner was off work for 12 days and went on to say, ‘That amount of time was hard because his was the only income coming in.’”
(O'Neil et al., 1988)	Lost income; lost land-based resource(s)	“Most women are the wage earners in families here. And so when a woman is on maternity leave, there’s no money coming into the family.” “Economic survival in northern communities is a formidable challenge and the loss of employment can have long-term implications on family health and well-being. Jobs and hunting are not alternative economic strategies but rather are interdependent. Without income, a man cannot maintain a snow machine or purchase gasoline and therefore cannot hunt. Loss of country food can have a devastating impact on the quality of a family’s diet.” “The economic costs to the family were also mentioned frequently. Hunting requires that a man be able to respond instantly to weather changes and information about caribou migrations, etc. It also requires considerable attention to the maintenance of equipment such as snowmobiles and sledges.”
(O'Neil et al., 1991)	Lost income; learning loss	“Loss of wages during pregnancy from employment is a significant problem for a sizeable minority of these women, as are the conditions of spousal employment where time and travel demands could create babysitting conflicts.” “Caregivers interviewed during the mother’s absence were asked to indicate difficulties encountered due to babysitting responsibilities. Many missed work or required other children to miss school to babysit.”
	Cost of childcare; cost of eldercare	“Many of these women have responsibilities for other children and some for elders that may create problems during pregnancy and evacuation for childbirth.”
	Cost of childcare	“While Inuit children spend a recognizable portion of their days and nights at homes of grandparents and relatives, this proportion increases substantially during their mother’s absence.”

### Intangible costs

Intangible costs were identified based on quotes from participants of the included studies. We grouped the intangible costs into themes, including fear, anxiety, depression, stress, isolation/loneliness, loss of connection to family and community, lack of respect for cultural practices, lack of support, compromised care, and lack of choice, control, and power. We honour the voices and experiences of the people who are impacted by obstetric evacuation (Table 4). In Brown et al. (2011), a participant summarized their experience by saying: “They said, you can be sent out, or you can be induced now… I had a choice, if you call that a choice.” The lack of choice, control, and power under the guise of accessibility was an underlying theme across several studies.

**Table 4:** Intangible costs of obstetric evacuation and transportation

Author, Year	Intangible cost	Quote
(Adams et al., 2021)	Fear; anxiety	“A fear of air travel was shared by many participants and deterred some participants from travelling for an ultrasound examination. A plane crash resulting in a fatality in the six months preceding the interviews remained on participants’ minds, and there was a general desire for residents to have their health-care needs met locally. Sometimes the fear of flying led participants to find other means of travelling to their appointments such as driving, even if the trip took 12 or 14 h.” “Depending on the time of their appointment, some patients stayed overnight in a hotel room provided through federal funding. However, these accommodations were often substandard: ‘But the accommodations were just gross, awful places to stay waiting for appointments and whatnot.... You know.... Who wants to stay in a dingy hotel like that, you know? When you live up here in a comfortable home where you feel at home, it’s just awful.’”
	Feelings of guilt; stress	“One participant described having felt guilty about having to expend government resources on travel for health services, resulting in delaying care: ‘For years of living here I felt guilty letting somebody else pay my way to P.A. [a city which has regular ultrasound services]. But that’s my treaty right. For years I’d just wait until I get to Regina to take care of my physical health needs. Because I felt guilty saying I need to go and have them pay.’”
	Isolation/loneliness	“Some obstetrical participants wanted to share the experience of having an ultrasound exam with their partner, but because of the need to travel and travel costs not being covered for their partner, found this was not possible: ‘And I was always alone going – I was told that I couldn’t bring my partner with me at the time to see the ultrasound. I don’t know why because it was some transportation thing they had to pay for. I don’t know.... all those three ultrasounds I went to I was there alone. [I felt] pretty upset because it was my first time pregnancy and it’d be nice for my partner to be there and actually hear the heart beat the first time and all that, yeah. I was pretty upset about that.’” “A larger city was an unfamiliar or strange place for many participants who had lived in a northern community their entire life. For obstetrical patients, especially at a younger age, going alone to a larger city was sometimes a frightening experience: ‘[The ultrasound exam] was in Saskatoon and I was just 18 so I never really travelled out alone that far so I was kind of scared. And my mom was so concerned about me when I went... And then after my ultrasound they didn’t tell me anything of what was going on with me; they just made me go back here.’”
(Brown et al., 2011)	Stress	“Pregnant and labouring women in each of the communities have to cope with — to varying degrees — hours of road and ferry travel and air medevac across open ocean and inlets in order to travel ‘up,’ ‘down,’ and ‘off’ island, ‘out’ and ‘away.’ Snow-covered roads and reduced daylight hours in winter added to the stressful circumstances for women leaving their home communities to give birth.”
	Isolation/loneliness; lack of choice, power, control	“The women described multiple ways in which their wants and needs were overridden: ‘I wasn’t allowed an escort’; ‘I was refused care’; ‘I was all alone’; ‘They said, you can be sent out, or you can be induced now… I had a choice, if you call that a choice.’” “The presence of family at birth was often a point of tension between hospital staff and women and their families. Women spoke repeatedly about efforts by hospital staff to limit visiting and their opposition to family presence, despite the support and understanding expressed by many health-care providers regarding the importance of family presence.”
	Stress; depression	“Contextual factors created significant emotional and financial burdens. One woman said, ‘Becoming poorer and poorer, having to live down island, and being alone is the absolute worst stress when you’re having a baby.’”
	Loss of connection to family and community; lack of respect for cultural practices	“In one community an Elder said: ‘There is a breakdown in the traditional family structure, as the mom is away from her community and family while she is giving birth. The family is excluded from the joy of being at the birth – this is important to our community, to our families.’” “Although nurses and physicians spoke of making birth ‘safe’ by having the women leave the community, women described the safety of their other children, and their own emotional safety surrounded by culture and connection to family and community, as overlooked in the structure and delivery of health care.”
	Fear	“A number of women expressed fear for the safety of the children they had to leave behind; even when they had reliable, safe child care, they worried about the impact of weeks or months of separation from their children and infants while they awaited birth, alone, elsewhere.”
(Cardinal, 2018)	Isolation/loneliness; stress; lack of support	“Imagine having to give birth in a foreign place by yourself and with no support system. Any medical escort or support person is expected to pay for their own travel and accommodation; few people living in remote areas have the financial means to do so. Most likely, you will end up sitting in a hotel room for a few days to a few weeks, then labour unaccompanied until the baby is born. Breastfeeding support may or may not be initiated by hospital staff. Sufficient emotional support may or may not be offered by hospital staff.”
(Chamberlain et al., 1998)	Lack of choice, power, control; stress	“For women who delivered outside the community, there were several issues relating to satisfaction, choices, wishes, help, and stress. All were dissatisfied with being away from their community for the birthing process and suffered stress as a result of being separated from their children. Most felt they had no choice in their place of delivery, birth procedures, or postpartum support.”
(Chamberlain & Barclay, 2000)	Isolation/loneliness; lack of respect for cultural practices	“The most frequently mentioned stressor was the enforced separation from family, culture and the community as a result of being sent out for a birth. Mothers were concerned for the well-being of children left behind.”
(Chamberlain et al., 2001)	Lack of choice, power, control	“Women who delivered out of their community felt they had little choice about moving around and positioning during labour and delivery.”
	Isolation/loneliness; lack of support	“Some mothers missed the support of partners during childbirth. Two women were homesick while two were bored waiting for the birth. These feelings were aggravated by the difficulties of living in residences with strangers, an unfamiliarly high environmental temperature and unfamiliar food.”
(Chatwood, 1996; Chatwood-Affleck et al., 1998)	Isolation/loneliness; lack of respect for cultural practices; stress	“By removing childbirth from a woman’s home environment, it has become an isolated event for Inuit women. Traditions which were once passed from Inuit midwives and mothers are being forgotten.” “They [Inuit women] miss their families and worry for the welfare of the children left behind.”
(Cooper et al., 2021)	Isolation/loneliness; compromised care; lack of respect for cultural practices	“I haven’t been home since January.... What makes it harder is I have three other kids at home.” “I don’t think people are getting the full services who actually need them. It’s not a luxury, it’s just simple basic services that are needed.” “I know that the health professionals are always helping us with our needs, but when it comes to low-risk birthing, those of us birthing at home both in Iqaluit and in the communities, those of us are not given the opportunity to practise our Inuit ways of birthing.”
(Kornelsen & Grzybowski, 2005)	Isolation/loneliness; compromised care	“Having to leave the local community to give birth seemed ironic to women for whom a sense of security was intimately associated with the network of support their local community provided.” “Many women felt that this lack of continuity and the consequent difficulty in forming relationships with care providers undermined the consistency of their maternity care.”
(Kornelsen & Grzybowski, 2006)	Fear; anxiety	“That was the only time I was a little concerned about being out here and being pregnant. The roads [are] bad. After the last ultrasound, I did come off the road in the snow, so that was a concern.”
	Stress; depression; loss of connection to family	“Anxiety over travel was not limited to getting to the referral hospital. Several participants noted concern about traveling back home with an infant, especially when the experience of motherhood was new.” “You know, the first time around, I think you’re prepared [to leave the community] because you don’t have any other responsibilities, but when it’s your second or third and you’ve got other children, that’s the hardest thing….My family’s not here so I don’t have….I can’t phone my mom and say, ‘Okay, can you come and watch the kids?’”
	Compromised care	“I’ve had postpartum depression...and I knew it was just because of the situation of being away from [my son] and everything…” “So then you have to go to [the referral community] once a week. I chose not to go because it was winter….So it was kind of tough because I got sort of flak for not going.”
(Lawford et al., 2018)	Lack of choice, power, control	“When asked if the women could go to Winnipeg for birthing services, Chief Merrick responded, ‘They don’t have that choice. Cross Lake goes to Thompson to give birth. Norway House goes to Winnipeg to give birth, right?’”
	Isolation/loneliness	“And then they ship them out to the city [alone] for... 2 weeks or 3 weeks before they’re actually supposed to give birth. And then they’re away from... their family for that long....to come and wait in the city to have a baby. Why can’t they wait at home?”
(Lawford et al., 2019)	Isolation/loneliness	“And I know this one lady was sent out, and she said, ‘Okay, I’ll go and wait.’ And she waited like a week and she got … She got lonesome… So she decided just to go home…. She came back and just went home. She didn’t want to wait in the city to have her baby. She wanted to be at home.”
	Compromised care	“Poor or absent communication on the part of health care providers with women and other care providers results in maternity care services that are uncoordinated and, as a result, far less than optimal.”
(O'Driscoll et al., 2011)	Isolation/loneliness	“Participants were lonely and missed the families they had left behind.”
(O'Neil et al., 1988)	Isolation/loneliness; anxiety; fear	“Although accommodation is provided in Inuit hostels in both Churchill and Winnipeg, women complain of loneliness, boredom, anxiety and fear.”
(Van Wagner et al., 2007)	Isolation/loneliness	“Air Inuit, the only regional airline, offers reduced airfare for partners to travel with women for childbirth. This is markedly different than the loneliness and disruption of evacuation to southern hospitals.”
(Varcoe et al., 2013)	Isolation/loneliness; loss of connection to family and community; lack of respect for cultural practices; compromised care	“In one focus group all seven young mothers cried through most of the interview, describing the effects of giving birth outside their communities: loneliness, disconnection from community, isolation from family and culture, and discrimination.”

## Discussion

Our systematic review of the literature identified 19 studies that met the inclusion criteria, and reported costs associated with evacuation or transportation to reach medical care. Direct, indirect, and intangible costs were extracted, based on the CoI approach. This study is the first we are aware of to systematically identify the costs of the obstetric evacuation policy across Canada. Our findings add to the body of research that has repeatedly demonstrated the medical, social, and psychological harms of evacuating Indigenous Peoples to reach obstetric care.

Existing literature has addressed the impact of the policy and described the experiences of those impacted. Silver et al. conducted a scoping review summarizing both factors and outcomes related to obstetric evacuation across Canada. Similar to our study, Silver et al. present quotes from participants, which in our study we compile to inform intangible cost themes. Our study further contributes to the literature by systematically reviewing the evidence related to costs and presenting the findings through a cost analysis framework. By reporting the direct, indirect, and intangible costs associated with evacuation, we present a quantitative and qualitative summary of the costs associated with obstetric evacuation across Canada.

Related research has also been conducted in countries with similar approaches to obstetric care, such as Australia. Gao et al. found that a Birthing in Our Community (BiOC) approach for First Nations Australians was associated with better outcomes and presented cost savings of AUD $4,810 per mother-baby dyad (Gao et al., 2023). The per-pregnancy travel costs were AUD $696 and AUD $176 for BiOC travel and standard care travel, respectively. The cost savings identified by Gao et al. are attributable to the reduced proportion of preterm birth in the BiOC group compared to the standard care group, rather than savings in travel costs. The generalizability of these travel costs to Canadian contexts is limited; however, it demonstrates the importance of context-specific descriptive cost assessment as it can inform economic evaluations. Along with existing research, and consultation with impacted communities, economic evaluations offer an additional perspective that can inform the implementation of alternative approaches to delivering obstetric and maternal-child care.

Our review assessed reported direct, indirect, and intangible costs of obstetric evacuation and transportation. The included studies indicated that funding of obstetric evacuation‒related services was shared among different types of payers, including—but not limited to—federal, provincial, and territorial governments as well as individuals paying for costs out of pocket.

Direct costs varied, given the range of contexts of when and where obstetric evacuation had taken place. The lowest reported cost of medical evacuation was $7,714 with the figure reaching a high of $31,794. Variation in cost is likely due to travel distance. All reported medical evacuation costs involved a form of air travel, and were typically reported from remote northern communities. Grouped costs of transportation and accommodation also exhibited a large range.

Indirect costs represent an important cost category, as they account for costs associated with land-based resources and community-based care networks. Indirect costs (Table 3) included lost income, cost of childcare, cost of eldercare, lost land-based resources or income, and learning loss. Learning loss was described as children missing school when taking on childcare responsibilities. Productivity costs of missed time from school have been quantified in the broader literature, but no quantified costs were reported by the included studies for the aforementioned indirect costs (Chari et al., 2015; Goldstein et al., 2014). For rural and remote Indigenous communities, these costs represent a substantial component of the overall economic loss. The government of Nunavut approximates the traditional harvesting (or land-based economy) to be worth $40 million annually, with the replacement food value of seal meat alone reaching approximately $5 million (Government of Nunavut, 2023; Vail & Clinton, 2001). Missed hunting to accompany a pregnant mother or to stay home and take care of children represents substantial costs. Other costs that were explored in a limited capacity included learning loss, whereby “Many [patients] missed work or required other children to miss school to babysit” (O’Neil et al., 1991). These indirect costs can have long-term impacts, with accumulating economic costs that have not been explored previously. Indirect and intangible costs have been especially overlooked when reporting medical evacuation and transportation costs.

Intangible costs have definitive value; however, they have not been assigned a monetary cost in the context of obstetric evacuation. As such, we included these costs (Table 4) reflecting the reporting approach of the respective review studies. The intangible costs were sorted into themes, including fear, anxiety, depression, stress, isolation/loneliness, loss of connection to family and community, lack of respect for cultural practices, lack of support, compromised care, and lack of choice, control, and power. The reporting of these costs is eerily consistent across the decades of published and grey literature. This is an indication of stagnation with regard to updating, improving, or even assessing the impacts of obstetric evacuation by policy makers and governments.

Historic context of colonialism is the foundation of existing approaches to health care delivery in Canada. This is exemplified in the intersection of the Canada Health Act and the Indian Act, which present unique and compounded challenges––and costs––for health care delivery in Indigenous communities. In this paper, we highlight the extensive evidence base that should inform policy making but has not been considered or implemented meaningfully. The federal government should account for the evidence base in their decision-making and assessment of obstetric care funding and delivery for First Nations and Inuit in Canada. Rebuilding culturally safe Indigenous birthing care is multifactorial and will require significant investments in recruitment, education and training, payment for services, and building care facilities. Our study investigates the cost of obstetric evacuation, and we view this as the first step to understanding how existing resources are being spent. Our future goals are to conduct economic evaluations comparing culturally safe practices, such as Indigenous midwifery, to obstetric evacuation. Although obstetric evacuation can be an appropriate approach in certain circumstances, it should be critically assessed why it has become the de facto approach to accessing obstetric care. Indigenous knowledges of healthy birthing can show the way forward for Indigenous childbearing women. This knowledge, combined with the resilience and resistance of First Nations, Inuit, and Métis, must be recognized at the institutional level by governments and health care systems and on an individual level by non-Indigenous Peoples in Canada. Efforts to return birth to Indigenous communities, by Indigenous midwives and community members, has a long-standing history. The National Council of Indigenous Midwives (NCIM) and Pauktuutit, both partners of this research effort, advocate for return of birth to community and betterment of maternal-child health. Indigenous-led models of care have existed for centuries and continue to demonstrate successful outcomes today. For example, the Inuit-led, community-based Inuulitsivik midwifery service located in Nunavik region of northern Quebec has been a model of birthing in community for decades (Van Wagner et al., 2007, 2012). Beyond clinical measures of success, championing Indigenous midwifery aligns with the Truth and Reconciliation Commission’s Calls to Action, as highlighted by the NCIM: “Increasing the number and capacity of Indigenous midwives fulfills the Truth and Reconciliation Commission’s Calls to Action to recognize the value of Indigenous healing practices, and to increase the number of Indigenous professionals working in the health care field” (National Council of Indigenous Midwives (NCIM), 2023).

### Limitations

Some databases limited the word count of the search strategy, restricting the number of terms included. Therefore, the search strategy did not include the names of specific First Nations or Inuit communities, leading to a less comprehensive search. Outdated and offensive terms were included, to ensure research from previous decades was not excluded. This does not condone any of the terms or language used historically, but ensures relevant studies were captured. Additionally, the precision of reported costs was another limitation of the review. The majority of reported costs were either average per diems or based on expert opinion. Costs provided by expert opinion included organizations such as MedEvac Canada or individuals such as the Director of the Kivalliq Regional Health Centre in Rankin Inlet. Some of the costs were patient reported. This further highlights the gaps in knowledge and understanding of the economic costs of obstetric evacuation.

The relevant policies provide insights into the processes involved in obtaining payment for health services. Costs incurred by federal or provincial/territorial governments are processed through relevant health plans or programs, such as NIHB and Nunavut Health Care Plan, respectively. Section 4.4 of the NIHB Medical Transportation Policy Framework states: “Medical transportation benefits for emergency ground ambulance include only the portion of the services not covered by provincial or territorial health or social programs, other publicly funded programs or private health insurance plans.” A similar approach applies to medical transportation benefits for emergency air ambulance or medevac services as stated in Sect. 4.5 of the policy (Indigenous Services Canada, 2020).

Overall, the costs identified were not comprehensive. We did not find estimates of a range of relevant costs. Even if a cost is ultimately covered by one or more levels of government, individuals are required to pay out-of-pocket at times. Several opportunity costs were not quantified, including costs incurred while waiting for reimbursement, cost of time spent navigating the payment, cost of time spent arranging travel for care, and the costs associated with avoidance of care due to complicated mechanisms.

Our systematic review was focused on travel for care and thus was not targeted towards identifying the costs of providing care in urban centres. The costs of care, which may be increased due to the discontinuity of care and lack of mechanisms for sharing health information between community health infrastructure and urban hospitals, may result in duplication of tests, poor quality of care, adverse health effects, and additional economic costs to individuals, families, and the health care system. The cost of providing culturally safe care in urban centres was also not a target of the study but a potentially relevant cost.

## Conclusion

Tackling the colonial roots of Health Canada’s policies, specifically obstetric evacuation, and assessing the historic and contemporary impact will contribute to Canada’s path towards truth and reconciliation. The assessments of costs, and ultimately economic evaluations, can contribute insights supporting existing efforts of Indigenous communities and organizations to return birth to communities, ensure care delivery is culturally safe, and reduce the number of medical evacuations. This study demonstrated that Indigenous-specific experiences of health care are often not captured in Canadian national data. The themes emerging from the systematic review show that obstetric evacuation costs are higher than previously estimated. Indigenous women’s experiences shed light on the complexity of indirect and direct costs that often go uncounted.

## Contributions to knowledge

What does this study add to existing knowledge?

- Our study further contributes to the literature by systematically reviewing the evidence related to obstetric evacuation costs and presenting the findings through a cost analysis framework.
- By reporting the direct, indirect, and intangible costs associated with evacuation, we present a quantitative and qualitative summary of the costs associated with obstetric evacuation across Canada.

What are the key implications for public health interventions, practice, or policy?

- There is a continued need for researchers to inspect costs and provide accurate measures, ensuring the nuances of community, geography, and policy are taken into account.
- Further research can inform effective practice policy that prioritizes Indigenous birthing knowledge and the experiences of Indigenous women in obstetric care.

## Data Availability

Study protocol available from https://www.crd.york.ac.uk/prospero/display_record.php?ID=CRD42022353800 (PROSPERO 2022 CRD42022353800).

## Appendix. Search strategies

### Search strategy (default)

(Indigenous OR Aboriginal OR Metis OR Inuit OR “First Nation*” OR “American Indian” OR “Native American Indian” OR Native OR Eskimo) AND (Canada OR Alberta OR “British Columbia” OR “Manitoba” OR “New Brunswick” OR “Newfoundland and Labrador” OR “Nova Scotia” OR “Ontario” OR “Prince Edward Island” OR “Quebec” OR “Saskatchewan” OR “Northwest Territories” OR “Nunavut” OR “Yukon”) AND (Rural OR remote OR isolate* OR evacuation OR transport OR transfer OR relocation OR travel OR “medically underserved area” OR “low resource environment”) AND (Maternity OR prenatal OR childbirth OR postnatal OR pregnan* OR obstetric OR delivery OR reproduct* care OR perinatal) AND (Human OR female OR women OR mother OR two-spirit OR “trans* man”).

### Search strategy (ScienceDirect)

(Indigenous OR Aboriginal) AND (Canada) AND (rural OR remote) AND (“obstetric evacuation” OR childbirth).
